# Circadian-like patterns in electrochemical skin conductance measured from home-based devices: a retrospective study

**DOI:** 10.3389/fneur.2023.1249170

**Published:** 2023-10-26

**Authors:** Benjamin Vittrant, Violaine Courrier, Rui-Yi Yang, Paul de Villèle, Samuel Tebeka, Sibylle Mauries, Pierre A. Geoffroy

**Affiliations:** ^1^Withings, Issy-les-Moulineaux, France; ^2^Département de Psychiatrie et d'addictologie, AP-HP, GHU Paris Nord, DMU Neurosciences, Hôpital Bichat—Claude Bernard, Paris, France; ^3^Centre ChronoS, GHU Paris—Psychiatry & Neurosciences, Paris, France; ^4^Université Paris Cité, Diderot, Inserm, FHU I2-D2, Paris, France

**Keywords:** electrochemical skin conductance, chronobiology, Withings, time series, connected scale

## Abstract

In this study, we investigated the potential of electrochemical skin conductance (ESC) measurements gathered from home-based devices to detect circadian-like patterns. We analyzed data from 43,284 individuals using the Withings Body Comp or Body Scan scales, which provide ESC measurements. Our results highlighted a circadian pattern of ESC values across different age groups and countries. Our findings suggest that home-based ESC measurements could be used to evaluate circadian rhythm disorders associated with neuropathies and contribute to a better understanding of their pathophysiology. However, further controlled studies are needed to confirm these results. This study highlights the potential of digital health devices to generate new scientific and medical knowledge.

## Introduction

The digital revolution has transformed the medical profession in numerous ways. It has introduced new concepts such as virtual reality (1–3) and has enhanced existing practices through telemedicine (4–7). These advances enable physicians to remotely monitor patients, saving them valuable time to focus on other aspects of their work. The use of these digital technologies is constantly growing, producing new scientific and medical knowledge by offering larger and more objective data collection (8). Indeed, it increases the number of data points compared to classical one-shot measures from follow-up at the hospital or other medical structures. While this raises concerns about data quality (9), legal considerations (10), and security (11), it has already proven its utility in various fields like clinical trials (12–16).

Withings (17) recently integrated into two scales (Body Comp & Body Scan) the electrochemical skin conductance (ESC) measurement previously featured into the Sudoscan (™) device (18–20), bringing a hospital-grade measuring technique to people’s homes. The measure of ESC can be used for the diagnosis of small-fiber neuropathy (SFN), which affects thinly myelinated and unmyelinated small-diameter peripheral nerve fibers mediating sensory or autonomic function. Alteration of thermal or nociceptive sensations and dysautonomia associated with SFN usually results in a negative effect on a patient’s quality of life (21–24). Since the ESC is a direct stimulation and not a recording of arousal stimuli it has been demonstrated to be very repeatable (19, 25–27). This led to its adoption in specific cases of diabetes grading and follow-up (28, 29).

Integrating ESC measurement into a connected scale gave us access to a wide range of real-world data at a scale that had never been reached before. This integration, along with previous research (30, 31), led us to set up our first objective: Can we identify the daily circadian rhythm of ESC (31) on a large scale under real-life conditions using a scale (Body Comp & Body Scan), in the context of SFN diagnosis that has already been conducted in a hospital setting (Sudoscan)?

Positive results would motivate us to set up a clinical study to have a better understanding of the individual profiles of ESC throughout the day. It would also help us to compare normal profiles vs. neuropathic profiles to understand how SFN may affect circadian patterns of ESC and allow us to integrate chronobiology as a biomarker if associated with any condition.

## Materials and methods

### Dataset

To build our dataset, we selected a subset of users of the Withings Body Comp and Body Scan scales, which are the devices providing the ESC measurements. We selected only patients aged 18 years and over with a minimal weight of 30 kg. The minimal weight is a threshold to exclude measures taken by young children using adult accounts. Then, we summarized our measurements at the minute level, meaning that if one individual performed several measurements within the same minute, then all the results were regrouped as one average. Returned, defective, and factory QA samples were also removed to avoid technical outliers.

The conditions we applied for data selection led us to keep 1,905,580 measures from 43,284 individuals, including 26,915 men and 16,369 women, from Germany—DE (34%), United States—US (25%), France—FR (21%), United Kingdom–GB (11%), Switzerland—CH (6.8%), and Austria—AT (3%). The average age of our population was 46.8 (13.9). Details can be found in Table 1 at the measures and individual levels. Since the data originated from the real world (as opposed to a clinically controlled study), they were sparse and imbalanced, and some individuals had more measurements than others. Older people tend to had more measurements than younger people in general, but no major imbalance was observed between measurements and individuals except for that small observation.

**Table 1:** This table presents the information (age, country, and gender) we have about our measures and individuals separately.

		Measures			Patients		
		Overall	Man	Woman	Overall	Man	Woman
*N*		1,905,580	1,397,559	508.021	43.284	26.915	16.369
Age		51.0 (13.3)	50.9 (13.4)	51.1 (13.0)	46.8 (13.9)	47.0 (14.0)	46.5 (13.7)
Age group							
	*Under 30*	119,555 (6.3%)	88,590 (6.3%)	30,965 (6.1%)	5,515 (13%)	3,409 (13%)	2,106 (13%)
	*30–39*	324,116 (17%)	238,947 (17%)	85,169 (17%)	9,457 (22%)	5,673 (21%)	3,784 (23%)
	*40–49*	460,732 (24%)	341,956 (24%)	118,776 (23%)	10,696 (25%)	6,693 (25%)	4,003 (24%)
	*50–59*	535,653 (28%)	387,049 (28%)	148,604 (29%)	10,337 (24%)	6,469 (24%)	3,868 (24%)
	*60–69*	325,174 (17%)	235,193 (17%)	89,981 (18%)	5,240 (12%)	3,328 (12%)	1,912 (12%)
	*Over 70*	140,350 (7.4%)	105,824 (7.6%)	34,526 (6.8%)	2,039 (4.7%)	1,343 (5.0%)	696 (4.3%)
Country							
	*AT*	57,545 (3.0%)	44,456 (3.2%)	13,089 (2.6%)	1,303 (3.0%)	816 (3.0%)	487 (3.0%)
	*CH*	117,159 (6.1%)	91,187 (6.5%)	25,972 (5.1%)	2,957 (6.8%)	1,874 (7.0%)	1,083 (6.6%)
	*DE*	690,929 (36%)	511,915 (37%)	179,014 (35%)	14,531 (34%)	9,164 (34%)	5,367 (33%)
	*FR*	305,950 (16%)	227,448 (16%)	78,502 (15%)	9,011 (21%)	5,523 (21%)	3,488 (21%)
	*GB*	172,980 (9.1%)	131,025 (9.4%)	41,955 (8.3%)	4,593 (11%)	2,912 (11%)	1,681 (10%)
	*US*	561,017 (29%)	391,528 (28%)	169,489 (33%)	10,889 (25%)	6,626 (25%)	4,263 (26%)

For age, the (SD) is also given. The countries are Germany (DE), the USA (US), France (FR), the United Kingdom (GB), Switzerland (CH), and Austria (AT).

The overall values of ESC are presented in Table 2. We observed decreasing values with age and gender, but no observable differences between countries in general, which was expected as reported in previous studies (32, 33).

**Table 2:** This table presents the overall and group-specific mean (SD) values of the ESC within our users.

			Overall										
	ESC		*62.2 (19.1)*										
Gender			Men					Women					
	ESC		*63.1 (19.2)*					*59.8 (18.8)*					
Age			40−	40–49	50–59	60–69	70+		40−	40–49	50–59	60–69	70+
	ESC		*68.0 (18.2)*	*63.9 (18.7)*	*61.3 (19.2)*	*59.7 (19.3)*	*56.9 (19.6)*		*65.7 (17.9)*	*60.1 (18.3)*	*58.2 (18.5)*	*56.0 (18.6)*	*53.0 (19.3)*
Country		AT	CH	DE	FR	GB	US	AT	CH	DE	FR	GB	US
	ESC	63.4 (18)	62 (18.3)	61.6 (18.9)	61.7 (18.9)	64 (19)	62.7 (20.4)	59.8 (16,9)	59 (18.1)	58.8 (18)	58.7 (18.4)	60.7 (18.4)	58.7 (19.9)

### Measuring devices

Electrochemical skin conductance (ESC) is a physiological measurement that can reflect the conductance of the sweat glands in the skin. The Withings Body Scan and Body Comp scales are non-invasive medical devices designed to be used at home daily to assess sweat gland function and diagnose autonomic neuropathies. They measure the electrochemical conductance of the feet, providing insights into the integrity of the autonomic nervous system. They have multiple functions (weight and pulse wave velocity), are approved by the U.S. Food and Drug Administration (FDA; K230912), and have medical CE approval (ECM22MDR001). Data were collected by our application and then sent to a specific medical cloud in accordance with the legal constraints of each country.

The technique is based on the principles of reverse iontophoresis and chronoamperometry, similar to Sudoscan (34). The device includes stainless steel plates on which the patients place their feet. These electrodes deliver a low DC voltage (less than 4 V), which activates the sympathetic innervation of the sweat glands, producing an outflow of chloride ions, which is the origin of a current generated by an electrochemical reaction and which will be measured at the level of the electrodes. Thus, the electrodes serve both as stimulation and recording electrodes. The conductance (ESC) corresponding to this current induced by the chloride ions is expressed in microSiemens (μS) and is objective and quantified data that directly reflects the magnitude and activity of the innervation of the sweat glands by unmyelinated C fibers (18).

This approach distinguishes itself from traditional electro dermal activity (EDA) measures such as skin conductance response (SCR) (35, 36) by directly stimulating the sweat gland directly with specified voltage steps. It is a direct stress test and not a continuous measurement in response to arousal stimuli. The full technical details can be found in the study by Khalfallah et al. (18).

### Visualization and statistical analysis

The data exploration was performed using R v4.2.3 (37), RStudio Build 386 (38), and Quarto v1.2.^1^ The plots were built using the ggplot v3.4.2 (39) and the ggpubr v0.6.0 (40) packages, and all statistics were performed using the easy stats v0.6.0 (41) package. Since we had many samples, normality tests were performed using the Anderson-Darling test instead of the classical Shapiro–Wilk one. To test mean differences, we used a *t*-test, while circadian patterns were checked using the Circacompare V0.1.1 package (42). This package quantified the circadian rhythm by fitting a periodic regression using a mean basis parameter, a 24-h cosine period, and other parameters.

### Generative AI

Since the latest development of large language models (LLM), generative AI has started to become an important tool for various research jobs. To follow the ethical guidelines about that subject, we will state how we used it here. In our case, we used gpt-4 (43) to write the abstract of the publication after feeding the prompt with our full text. All the other content was human-generated.

The following paragraph is the text that was given to ChatGPT to generate the abstract after feeding it the full publication (excluding the abstract):

“*I want you to act as a lead scientist working in neuropathies medical research. Your team wrote a publication and they need you to write the final abstract. I’m going to give the full publication. There are no figures and tables, including only their associated legends. The abstract should be less than 300 words and understandably summarize the work.*”

### Ethical consideration

Data were collected within the device’s intended use without any action from Withings toward the individuals; thus, our study is neither interventional nor clinical. No individual or medical data from specific individuals was presented in this study, and all the data were aggregated, making them non-identifiable. For the data presented individually, we did not provide the age, gender, or data localization, making them also unidentifiable.

## Results

**Figure 1:**
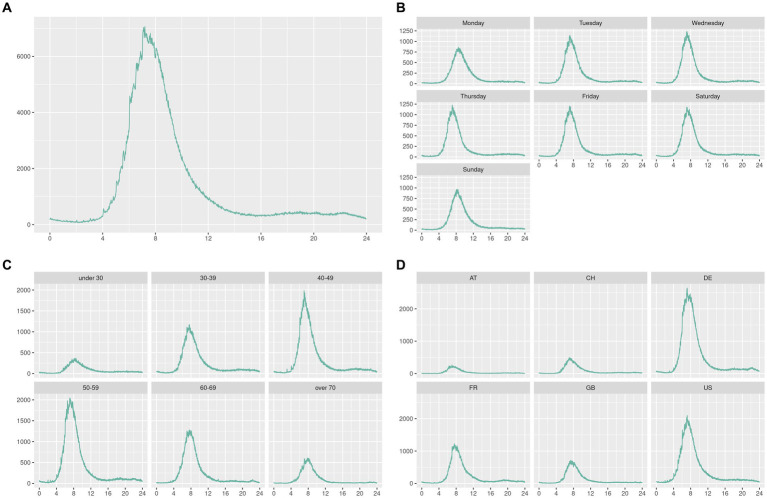
In this graph, we plotted the measures distribution throughout the day (0–24 h) for all the data in **(A)** and then split them by day **(B)**, age **(C)**, and country **(D)**.

We first checked how the data were globally distributed within the day (Figure 1A). Then, we checked that individuals had the same behaviors of taking measurements during the week and the weekend while being homogeneous across age groups and countries (Figures 1B–D). We did not observe any particular usage in any group that could have led us to isolate it from the analysis.

**Figure 2:**
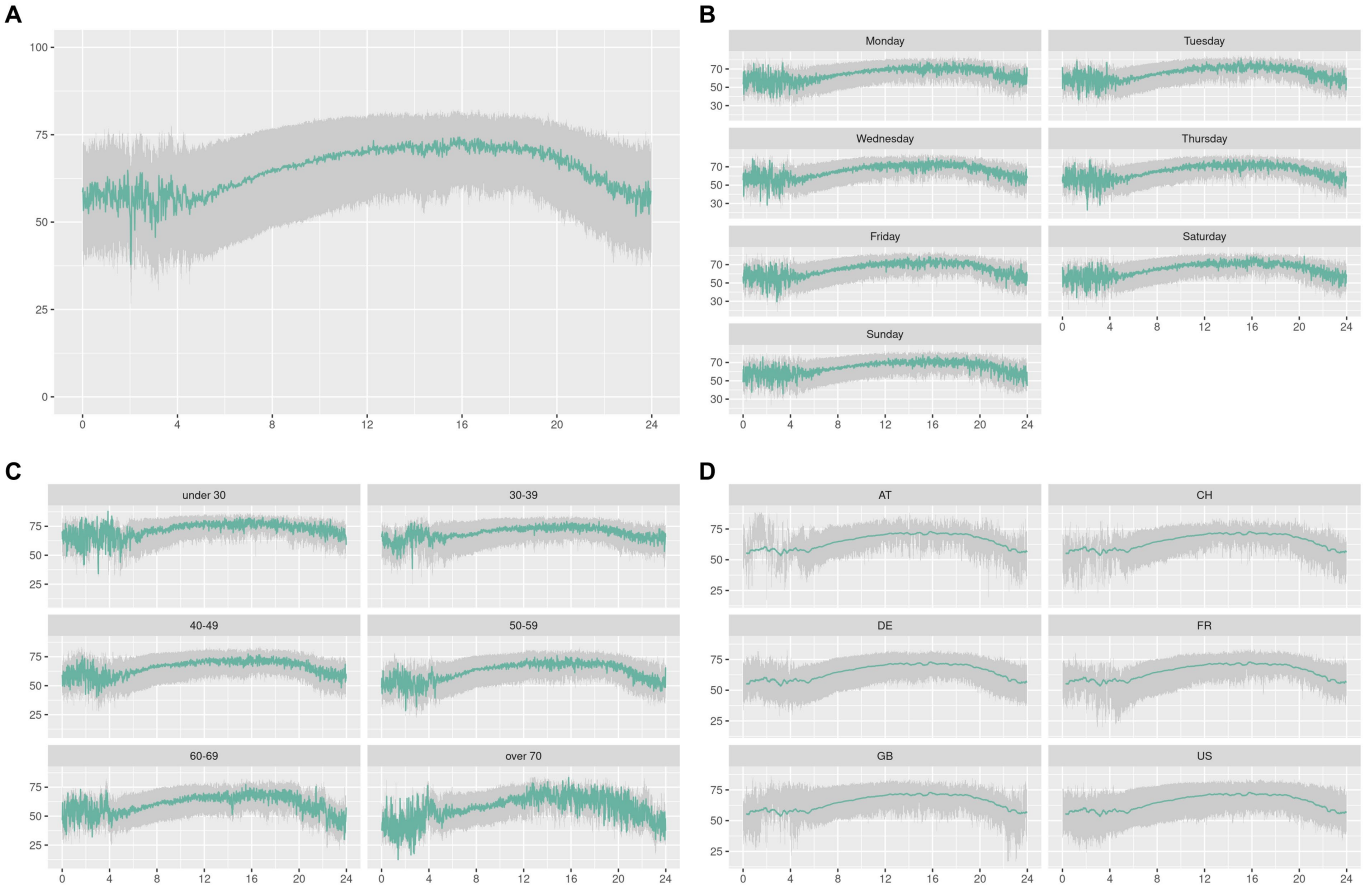
In this graph, we plotted the values of the ESC throughout the day (0–24 h) for all the data in **(A)** and then split them by day **(B)**, age **(C)**, and country **(D)**. The green curve is the median value and the lower/upper limit of the ribbon are the first/third quartile of the data. The countries are Germany (DE), the United States (US), France (FR), the United Kingdom (GB), Switzerland (CH), and Austria (AT).

Then, we checked the pattern of the median value throughout the day for all the data (Figure 2A) and throughout our different groups (Figures 2B–D). In this study, we observed a periodic pattern with an increase during the day and a decrease during the night. We also observed that late nightly values (23 h–4 h) had more variability since at the moment of the study we had not enough data to converge to a stable median.

**Figure 3:**
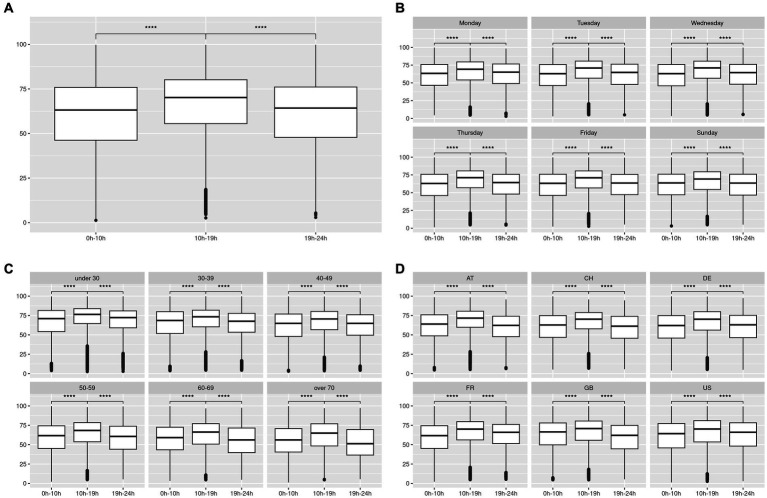
In this graph, we plotted the values of the ESC throughout our three defined time categories for all the data in **(A)** and then split them by day **(B)**, age **(C)**, and country **(D)**. T-test was performed using the 10–19 h category as a reference and the significance level was plotted over the boxplot. ^***^meaning results with value of *p* < 10e−3.The countries are Germany (DE), USA (US), France (FR), United Kingdom (GB), Switzerland (CH), and Austria (AT).

**Figure 4:**
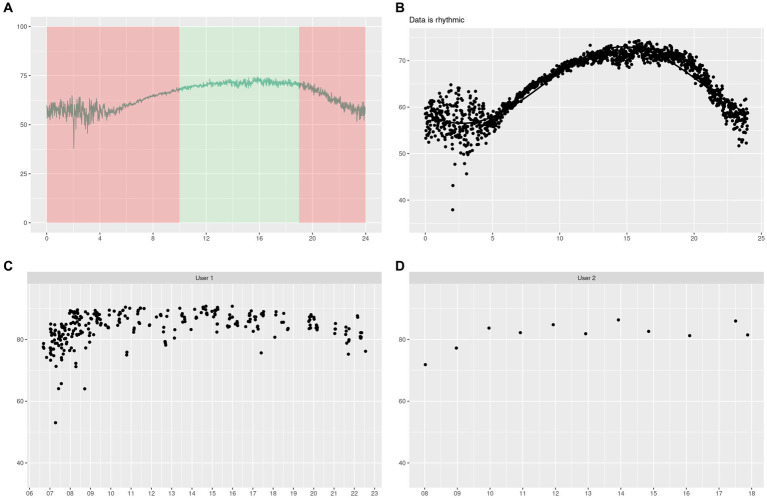
In this graph, we showed our defined green area of interest where values are at their maximum in **(A)** and the results of our circadian model in **(B)**. In **(C,D)**, we showed users’ data that had enough measures to illustrate the circadian pattern throughout the day at the individual level.

Next, we decided to split the ESC values by time categories to test whether there were differences between the daily values and the other time categories. We defined the time categories as 0–10 h (night-morning), 10–19 h (day), and 19–24 h (evening). We checked the normality of the data within our time categories (Anderson-Darling test, value of *p* < 2.2e−16) and then performed a *t*-test. The results are shown and plotted in Figure 3. Significant differences existed between the daily values and the rest across days, ages, and countries. This observation led us to set up a green zone (Figure 4A), in which the measurement should be taken if the value of interest is the maximum value the ESC can reach, or if the measure is compared with Sudoscan values (28, 44–46). Indeed, Sudoscan values were taken during hospital hours, therefore in our defined green zone. This green area shows the measurement’s stability between 10 am and 7 pm of ESC with values of median/mean/SD of 70.9/70.9/1.34 μS, which is in accordance with previous Sudoscan studies realized in hospitals.

Finally, we checked if our median values could be modeled by a cosine function (circadian pattern-like), and the results were positive (value of *p* < 1e−4) with a specific set of parameters:


$$ESC=k+\left(\mathrm{alpha}\right)*\;\cos\;\left(\left(1/\mathrm{period}\right)*\;time_{r}-\left(\mathrm{phi}\right)\right)\text{,}$$

where *k* = 64.713, *alpha* = 8.277, and *phi* = 3.860, and *time_r* is the time unit (e.g., hours and minutes), and the period is 24 h. The results can be checked in Figure 4B.

Following the global perspective, we decided to check on specific individuals who took many measures per day for several days and not around the same hour. As we worked directly on individual data, we did not have the luxury of getting many of them using the scale multiple times a day. Indeed, we only found two individuals (called *user 1* and *user 2*) who did that at the moment of data collection. They are illustrated in Figures 4C,D.

## Discussion

We first controlled that the individual behavior was similar between weekdays and weekends and also homogeneous across countries. Here, we did not observe any specific behaviors that could have led us to consider separating our analysis into distinct populations.

Then, we checked the ESC values and observed a pattern where ESC values started low in the morning, increased during the day to reach a plateau around midday, and then finally decreased in the late afternoon to night time. This pattern was also observed in different countries and on weekdays and weekends. More importantly, this pattern was also homogeneous across age categories and checked by a cosinusoidal model.

After splitting our data into morning, day, and evening time slots, we observed differences between daytime and other categories and confirmed that, on average, daily values were higher than morning and evening values. Finally, these results could be checked for two individuals with many measures that expressed the same daily pattern.

These results led to the conclusion that the Withings scales providing ESC measurements can be used to evaluate circadian rhythm disorders associated with neuropathies and to better understand their pathophysiology. More generally, it could be used with chronotherapeutics to help physicians objectively follow the effect of any therapy or treatment on their patients.

Our work is an observational study, and the results should now be verified with a more controlled case to draw definitive conclusions on our ability to follow circadian patterns from ESC measurements. Moreover, it brings up the question of pattern characterization in SFN. To our knowledge, no studies have been conducted since no measurement of ESC related to SFN is available in the form of individual time series except for our dataset. Thus, connected scales bring new and exciting opportunities for real-world evidence data and should be exploited along with clinical studies to bring the full medical potential of both together.

## Conclusion

Our results are encouraging, and integrating ESC measurement into widely used home-based devices opens up new possibilities for monitoring and understanding the link between SFN and circadian rhythms in real-world settings. Furthermore, the availability of real-world data collected through connected devices highlights the power of technology for generating new scientific and medical knowledge.

Future research should focus on further investigating the relationship between ESC, neuropathies, and circadian rhythms and exploring the potential clinical applications of ESC measurement as an objective circadian marker. Additionally, longitudinal studies are needed to examine how changes in ESC patterns over time relate to health outcomes and disease progression.

## Limitations

This study is purely an observational study with a large, one-sourced data set. It implies that there can be bias related to the Withings scales’ users while lacking medical information to stratify the population adequately. Therefore, we can only conclude on a general tendency, and we encourage researchers to test the ESC measurements within their medical fields to gain an understanding of the measurements and disease specificities.

## Data availability statement

The data analyzed in this study is subject to the following licenses/restrictions: data are available only for research purposes. Datasets are available on demand through our API (https://developer.withings.com/).

## Ethics statement

Ethical approval was not required for the study involving humans in accordance with the local legislation and institutional requirements. Written informed consent to participate in this study was not required from the participants or the participants’ legal guardians/next of kin in accordance with the national legislation and the institutional requirements.

## Author contributions

BV processed the data, created the graphics, and wrote the main part of the publication. VC, R-YY, PV, ST, SM, and PG provided corrections and meaningful insights and polished the writing. All authors contributed to the article and approved the submitted version.

## Footnotes

^1^ https://quarto.org/
